# Network and Phase Symmetries Reveal That Amplitude Dynamics Stabilize Decoupled Oscillator Clusters

**DOI:** 10.3390/e27050501

**Published:** 2025-05-07

**Authors:** Jeffrey Emenheiser, Anastasiya Salova, Jordan Snyder, James P. Crutchfield, Raissa M. D’Souza

**Affiliations:** Complexity Sciences Center and Physics, Mathematics, and Computer Science Departments, University of California, Davis, One Shields Avenue, Davis, CA 95616, USA; jemenheiser@ucdavis.edu (J.E.); jsnyd@uw.edu (J.S.); rmdsouza@ucdavis.edu (R.M.D.)

**Keywords:** synchronization, phase-amplitude oscillator, cluster stability, collective behavior, emergent symmetries, phase shift, nanoelectromechanical systems, ring network, symmetry breaking, isotropy subgroups

## Abstract

Oscillator networks display intricate synchronization patterns. Determining their stability typically requires incorporating the symmetries of the network coupling. Going beyond analyses that appeal only to a network’s automorphism group, we explore synchronization patterns that emerge from the phase-shift invariance of the dynamical equations and symmetries in the nodes. We show that these nonstructural symmetries simplify stability calculations. We analyze a ring-network of phase–amplitude oscillators that exhibits a “decoupled” state in which physically-coupled nodes appear to act independently due to emergent cancellations in the equations of dynamical evolution. We establish that this state can be linearly stable for a ring of phase–amplitude oscillators, but not for a ring of phase-only oscillators that otherwise require explicit long-range, nonpairwise, or nonphase coupling. In short, amplitude–phase interactions are key to stable synchronization at a distance.

## 1. Overview

Oscillator networks exhibit a wide variety of coordinated and collective behaviors, e.g., globally synchronized states, splay states, cluster states, and chimera states [1,2,3,4,5,6,7,8,9]. Stability calculations for cluster synchronization [9,10] and independently synchronizable clusters [2] were recently simplified using the underlying structural symmetries of the network connecting the oscillators. Yet, in addition to network connectivity, general symmetries may play a significant role in collective behavior. It is well known, for instance, that general dynamical systems exhibit emergent symmetries [11]. We adapt this insight to show that dynamical symmetries—phase-shift symmetries in nodal dynamics and coupling—are key to determining oscillator network stability. Most notably, these symmetries reveal collective states with nuanced behaviors—collective states that synchronize at a distance—whose stability properties can be understood using dynamical symmetries.

We focus, in particular, on a set of trajectories dubbed the “decoupled” state in a ring-network of phase–amplitude oscillators—a collective state whose existence was conjectured some time ago [12,13], but only recently realized experimentally [14]. This synchronization at a distance arises when oscillators, physically coupled along a nearest-neighbor ring network, begin to act independently of their immediate neighbors due to emergent cancellations in the dynamical equations of motion. The result is collective dynamical patterns that are higher-order and longer-range than the pairwise oscillator physical coupling. For instance, the decoupled state experimentally explored in Ref. [14] exhibits effective next-nearest-neighbor coupling in a network formed from purely nearest-neighbor physical coupling.

The decoupled state generally appears through symmetry breaking. To analyze stability we use the fact that, for each symmetry-breaking state, there is a corresponding subgroup of the system’s full symmetry group that leaves each component of that state invariant. The Jacobian, used to monitor the state’s stability, must commute with the subgroup symmetry operators at each point in the state’s trajectory [11]. This, in effect, defines “symmetry-breaking” by considering those symmetries of the dynamics that are not broken. Mutually diagonalizing these subgroup symmetry operators determines the corresponding block diagonal form of the Jacobian. If the block size is sufficiently small, we give the Jacobian’s eigenvalues and eigenvectors in closed-form. This tool allows us to investigate the stability of the decoupled state, even with arbitrarily large ring networks.

In this way, we establish that the decoupled state can be linearly stable for a ring of phase–amplitude oscillators, but not for a ring of phase-only oscillators with phase-based coupling (e.g., as in the Kuramoto model [4,15]). This demonstrates the importance of oscillator amplitude degrees of freedom for stability and for creating long-range effective couplings.

Our development proceeds as follows. First, we describe the oscillator network and its symmetry subgroups and show how these constrain the Jacobian. Second, we introduce a specific dynamic corresponding to the system experimentally studied in Ref. [14] consisting of a ring of nanoelectromechanical, phase–amplitude oscillators. Third, we show that symmetry considerations predict the existence of the decoupled state (similar to Refs. [8,12]) and, moreover, simplify the stability analysis. Fourth, when the decoupled state was achieved in experiment, drift in the phase difference between the decoupled clusters was observed [14]. We explain this by showing that small deviations in the oscillators’ natural frequencies break the symmetry and result in drift. Finally, with drift, the Jacobian becomes time dependent and so we use Floquet theory to analyze network stability. This analysis shows that alternating the natural frequencies of adjacent oscillators introduces an intricate dependence of the stability on natural frequency difference and other system parameters.

## 2. Symmetries and Stability

Consider a generic continuous-time dynamical system $\dot{x}=f\left(x\right)$ defined on a state space *X*. A set of invertible operations {γ:X→X}, together with operator composition, generates a symmetry group Γ of the dynamics if and only if, for all x∈X, each operation obeys:

(1) $$\begin{array}{c} f\left(\gamma x\right)=\left(D_{x}\gamma \right)f\left(x\right)\;, \end{array}$$

where $D_{x}\gamma :T_{x}X\to T_{x}X$ is the differential operation at *x*. This defines an *equivariant* dynamical system with respect to the symmetry group [16]. When the tangent (*T*) or differential (*D*) operators are independent of reference point *x*, we drop the subscript.

Under the restriction that each γ acts linearly on *X*, the differential operation becomes trivial, and the equivariance condition becomes: f(γx)=γf(x). This means one can side-step differences between *X* and TX. While such conditions are typically met by symmetry operations associated with the network structure, the nonlinear operations here require more careful handling. Similarly, curvilinear coordinates may disrupt the matrix representations of linear operations. In these cases, one must be mindful of the symmetry differences between the state space *X* and tangent space TX.

Given a dynamical system that respects a symmetry group Γ, the isotropy subgroups Σ≤Γ are those that leave state-space subsets invariant. A particular trajectory in state space, such as a fixed point or limit cycle, is said to respect an isotropy subgroup Σ if all points x(t) in the trajectory are invariant under Σ:

$$\begin{array}{c} \sigma x(t)=x(t)\;, \end{array}$$

for all σ∈Σ and all t∈R. For a given dynamical system, classes of trajectories may be predicted by identifying the system’s largest symmetry group and then identifying subgroups Σ that leave a nontrivial set of points in state space fixed. These state-space subsets are, themselves, dynamically invariant.

Σ’s group structure provides a convenient coordinate basis for describing the evolution of states close to the trajectory of interest and, therefore, for understanding its stability. In particular, we study the evolution of infinitesimal deviations x(t)+ϵδx(t), ϵ≪1. To leading order in ϵ, their evolution is governed by the linear ordinary differential equation:

$$\begin{array}{c} \frac{d}{dt}\delta x=J\left(x\right)\delta x\;, \end{array}$$

where $J=\frac{\partial f}{\partial x}$ is the system Jacobian.

Since the global evolution respects the symmetry operators σ, the linear dynamics respects symmetry operators Dσ(x). This means that the Jacobian and the symmetry operators commute at each point in the fixed-point subspace of Σ:

$$\begin{array}{c} D\sigma (x)J(x)=J(x)D\sigma (x)\;. \end{array}$$

The Jacobian, thus, shares eigenspaces with each of the differential group operators. To find these eigenspaces, we block diagonalize the matrices corresponding to the symmetry group generators. This can be done by finding the isotypic components of the differential group {Dσ:σ∈Σ}: each block corresponds to an irreducible representation of the group [11]. Since they commute, the linear operators Dσ(x) and J(x) then share Σ-irreducible invariant subspaces [17], acting on each subspace according to the corresponding diagonal block.

Below, we show how a behavior’s symmetry may be used to block-diagonalize the linear dynamics around any point of that trajectory class and so determine its stability properties. Assessing this class then reduces to two distinct problems: (i) evolving states within the subset of state space invariant to a known symmetry subgroup and (ii) evolving perturbations transverse to that subspace.

## 3. Phase–Amplitude Oscillator Ring

We now turn to the specific system under study—a ring of nanoelectromechanical (NEMS) phase–amplitude oscillators. For the experiments reported in Ref. [14], each oscillator is implemented as a piezoelectric oscillating membrane that is coupled with other oscillators electronically in a tunable network. The resulting oscillator networks exhibited a wide variety of exotic synchronization patterns including the decoupled state, mentioned above.

In fact, as found in Ref. [14], for a ring network the dynamical evolution is captured by well-understood equations of motion [18,19]. Representing the state of each of the *N* oscillators as a complex number $A_{j}$, with node index j=0,1,…,N−1, the dynamics are [13,14]:

(2) $$\begin{array}{cc} \frac{dA_{j}}{dt}= & -A_{j}+i\omega_{j}A_{j}+2i\alpha |A_{j}{|}^{2}A_{j}+\frac{A_{j}}{|A_{j}|}+i\beta \left(A_{j-1}-2A_{j}+A_{j+1}\right)\;. \end{array}$$

Indexing is taken modulo *N*. For the symmetry considerations discussed later, we limit our development to rings consisting of multiples of four (N=4M) oscillators.

There are three categories of tunable system parameters: $\omega_{j}$ is the natural frequency of oscillator *j*, α controls the Duffing nonlinearity (equal across all oscillators), and β is the reactive coupling strength between adjacent oscillators (equal across all pairs of neighbors). The dynamics of an individual oscillator in Equation (2) is similar to that of the widely-studied Stuart–Landau oscillator.

It is convenient to represent the state in $R^{2N}$, where each oscillator’s state is split into real amplitude and phase components: $a_{j}e^{i\phi_{j}}=A_{j}$. The equations of motion become:

(3) $$\begin{array}{cc} \frac{da_{j}}{dt} & =1-a_{j}-\beta a_{j-1}\sin\left(\phi_{j-1}-\phi_{j}\right)-\beta a_{j+1}\sin\left(\phi_{j+1}-\phi_{j}\right) \end{array}$$

(4) $$\begin{array}{cc} \frac{d\phi_{j}}{dt} & =\omega_{j}+2\alpha a_{j}^{2}+\beta \frac{a_{j-1}}{a_{j}}\cos\left(\phi_{j-1}-\phi_{j}\right)+\beta \frac{a_{j+1}}{a_{j}}\cos\left(\phi_{j+1}-\phi_{j}\right)-2\beta \;. \end{array}$$

We now consider this system’s symmetries; i.e., we identify the operations that satisfy Equation (1).

If all oscillators have *uniform* frequency, $\omega_{j}=\omega$, the ring dynamics respects the symmetry group generated by rotations $\sigma_{\mathrm{rot}}:A_{j}\mapsto A_{j+1}$ and by (node-centered) reflections $\sigma_{\mathrm{ref}}:A_{j}\mapsto A_{N-j}$ of the ring. The symmetry of the undirected cycle is called the *dihedral group* and denoted $D_{N}$. Its elements are the 2N unique products of $\sigma_{\mathrm{rot}}$ and $\sigma_{\mathrm{ref}}$. Elements of the dihedral group act as permutation matrices on both the complex coordinates of Equation (2) and the real coordinates of Equations (3) and (4), as is standard for topological symmetries. These operations merely reorder the nodal coordinates.

We also consider the case of *alternating* frequencies: $\omega_{j}=\omega \mp \Omega /2$, where even-numbered *j* follow the − and odd-numbered *j* the +, and we call Ω the *detuning*. (We will see that the mean frequency ω does not influence stability calculations and merely defines a frame of reference.) The network rotational symmetry is now reduced to that generated by $\sigma_{\mathrm{rot}}^{2}$. The reflectional symmetry $\sigma_{\mathrm{ref}}$ is still respected, generating a $D_{N/2}$ symmetry group.

Regardless of node frequency details, the ring also respects a continuous symmetry of uniform phase shifts: $\sigma_{\theta }:\left\{\phi_{j}\right\}\mapsto \left\{\phi_{j}+\theta \right\}$ with any 0≤θ<2π. We denote this continuous group T. In real amplitude and phase coordinates, this operation is affine yet nonlinear. Its differential $D_{x}\sigma_{\theta }$ is equivalent to the identity at all points *x*. This can be seen in Equations (3) and (4) by shifting all phases by θ: these phase shifts cancel and the equations of motion are invariant. However, in the complex amplitudes of Equation (2), this becomes the linear action $\sigma_{\theta }:\left\{A_{j}\right\}\mapsto \left\{e^{i\theta }A_{j}\right\}$.

Since uniform phase shifts commute with reordering node indices, the full symmetry group of the system is the direct product group $D_{N}\times T$ in the case of uniform frequencies (and $D_{N/2}\times T$ in the case of alternating frequencies). Subgroups of $D_{N}\times T$ may contain nontrivial phase shifts, revealing interesting synchronization patterns, as framed generally by Ref. [8] and used in Ref. [14] to characterize synchronization patterns in the NEMS system.

For each subgroup of system symmetries, the set of points left unchanged by the action of all symmetry operators defines an invariant set of the system dynamics. Time-evolution cannot break a symmetry of the initial condition, if the dynamics themselves respect that symmetry. To proceed, we identify a particular symmetry subgroup that defines an interesting invariant set in both the uniform and alternating frequency cases.

## 4. Decoupled Antiphase-Synchronized Clusters

**Figure 1:**
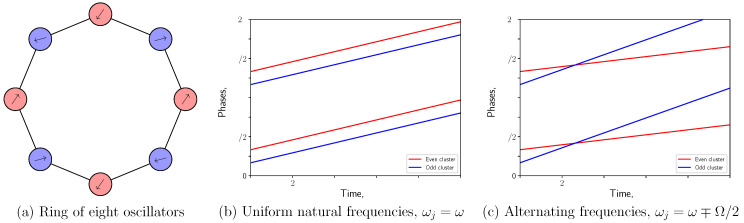
(**a**) Snapshot of a ring of eight oscillators in the decoupled state, with nodes colored according to synchronized cluster and arrows indicating local phase. Solid black lines indicate physical coupling between oscillators. (**b**) Reference phase of each decoupled cluster versus time for oscillators with uniform natural frequencies, demonstrating constant phase differences. (**c**) Reference phase of each decoupled cluster versus time for oscillators with alternating natural frequencies, demonstrating drift in phase difference between nodes in different clusters. The nonlinearity, coupling strength, and mean natural frequency are α=0.1, β=1.0, and ω=2, respectively. The difference in natural frequencies between the two clusters is (**b**): Ω=0 and (**c**): Ω=0.2.

With this background in mind, we turn to the collective state of interest—the decoupled state in which each node appears to act independently of neighbors to which it is physically coupled. For the equations of motion in Equation (2), independence is the condition that $A_{j-1}+A_{j+1}=0$; that is, next-nearest neighbors must be locked in perfect antiphase. This condition causes the coupling terms to cancel out, so that each oscillator appears to evolve under the influence of itself alone. On a ring of *N* oscillators, this is only satisfied if N=4M. The system then splits into a group of antiphase synchronized even-numbered oscillators and a group of antiphase synchronized odd-numbered oscillators, as illustrated in Figure 1a. Remarkably, there is no constraint on the phase differences between the two groups.

This decoupled state reflects a system symmetry. Note that the requirement $A_{j-1}+A_{j+1}=0$ is exactly the condition for a state to be invariant to the operation $\sigma_{\pi }\sigma_{\mathrm{rot}}^{2}$. This operator rotates the ring by two oscillators and advances the phase of all oscillators by one half period: $\sigma_{\pi }\sigma_{\mathrm{rot}}^{2}A_{j}=-A_{j+2}$. It generates a cyclic group of order N/2 and is a member of the symmetries of both the uniform frequency and alternating frequency cases. The decoupled states, such as the one shown in Figure 1a, lie precisely in the fixed-point subspace of this subgroup of the system symmetry and, therefore, are an invariant set of the dynamics.

This requirement also simplifies the coupling term in Equation (2) (i.e., the final term) to $-2i\beta A_{j}$. This means that the evolution of each oscillator is determined only by its own dynamic state. Using this in Equations (3) and (4) one sees that each oscillator amplitude will approach unity, behaving as if it were uncoupled.

An oscillator’s exact phase is arbitrary, as is the phase difference between the even- and odd-numbered oscillators. Defining a global reference phase θ and phase difference ψ, we obtain the set of solutions describing the decoupled state:

(5) $$\begin{array}{cc} a_{j}\left(t\right) & =1,\\ \phi_{j}\left(t\right) & =\left\{\begin{array}{cc} \theta (t) & \\ \theta (t)+\psi (t) & \\ \theta (t)+\pi & \\ \theta (t)+\psi (t)+\pi , \end{array}\;j\;\;\;\mathrm{mod}\;4=\begin{array}{c} 0\\ 1\\ 2\\ 3 \end{array}\right.\;. \end{array}$$

For both the uniform- and alternating-frequency cases:

$$\begin{array}{cc} d\theta /dt & =\omega +2\alpha -2\beta \\ d\psi /dt & =\Omega \;. \end{array}$$

Recall that Ω=0 for uniform frequencies and Ω≠0 for alternating frequencies, so ψ is fixed in time in the former case, but drifts according to the detuning Ω for the latter. Examples are shown in Figure 1b,c. The initial values of θ and ψ are free variables defining a 2-torus. The solutions over all possible initial values form a set of limit cycles that foliate the torus.

## 5. Stability

This particular decoupled state (with multiple anti-synchronized clusters) was previously studied via symmetry considerations, for instance, on weakly coupled identical phase oscillators [5,8] and linearly coupled oscillators [12,20]. Stability properties were not addressed. Recently, it was shown how a broad variety of decoupled states can arise from balanced equivalence relations rather than from symmetries and that symmetries are only required in an effective network of clusters of nodes [21]. However, Ref. [21] addressed only the stability properties of networks of uniform nodes. Here, we provide stability analysis in cases of both uniform and alternating frequencies and for a specific dynamics.

To set up the linear stability analysis of the decoupled state, we next use the symmetry structure to block-diagonalize the Jacobian. Given the solution in Equation (5) to the dynamics of Equations (3) and (4), the linear dynamics of real deviations to amplitudes δa and phases δϕ are:

(6) $$\begin{array}{cc} \frac{d\delta a_{j}}{dt} & =\left[\beta \sin\psi \delta a_{j-1}-\delta a_{j}-\beta \sin\psi \delta a_{j+1}\mp \beta \cos\psi \delta \phi_{j-1}\pm \beta \cos\psi \delta \phi_{j+1}\right] \end{array}$$

(7) $$\begin{array}{cc} \frac{d\delta \phi_{j}}{dt} & =\left[\pm \beta \cos\psi \delta a_{j-1}+4\alpha \delta a_{j}\mp \beta \cos\psi \delta a_{j+1}+\beta \sin\psi \delta \phi_{j-1}-\beta \sin\psi \delta \phi_{j+1}\right]\;, \end{array}$$

where perturbations to even- (odd-)numbered oscillators follow the upper (lower) ±/∓ option. This system of linear ODEs is independent of the global phase θ and depends on the phase difference ψ.

We simplify this linearization by finding convenient coordinates. With the solution contained in the fixed-point subspace of the cyclic group Σ generated by $\sigma_{\mathrm{rot}}^{2}\sigma_{\pi }$, we know that DσJ=JDσ for all σ∈Σ. Equivalently, through {Dσ}’s isotypic components and through the eigendecomposition of the generating Dσ, we find a coordinate system in which the Jacobian is block diagonal, consisting of N/2 blocks of size 4×4. First, we define $\zeta^{k}=e^{\left(4ki\pi /N\right)}$ to be the (N/2)th root of unity. This corresponds to the N/2 order of our cyclic group. Let us define vectors of length N/2: $\Phi_{j}^{\left(k\right)}=\zeta^{k\cdot j}$, with $k=0,1,\ldots ,\frac{N}{2}-1$. These $\Phi^{\left(k\right)}$ vectors are wave patterns over N/2 elements. Each element of a $\Phi^{\left(k\right)}$ is associated with one adjacent pair of oscillators, each with two real degrees of freedom. We therefore take the matrix outer product with two 2×2 identity matrices, $I_{2}$: the first for the pair of oscillators and the second for the amplitude and phase of each. This creates the 2N×4 matrices $V^{\left(k\right)}=I_{2}⊗\Phi^{\left(k\right)}⊗I_{2}$. These matrices, in fact, define 4 eigenvectors of the generating (and therefore every) group operator. The columns share an eigenvalue: $\sigma_{\mathrm{rot}}^{2}\sigma_{\pi }V^{\left(k\right)}=\zeta^{k}V^{\left(k\right)}$.

Since $V^{\left(k\right)}$’s columns exactly span an eigenspace (isotypic component), the Jacobian may be written in a coordinate system in which it is block diagonal with 2M blocks of size 4×4, with each block specified by $D_{k}=V^{(k)T}JV^{\left(k\right)}$:

(8) $$\begin{array}{cc} \; & D_{k}=\frac{1}{2}\left[\begin{array}{cccc} -1 & -\beta (1-\zeta^{-k})\sin\psi & 0 & \beta (1-\zeta^{-k})\cos\psi \\ \beta (1-\zeta^{k})\sin\psi & -1 & \beta (1-\zeta^{k})\cos\psi & 0\\ 4\alpha & -\beta (1-\zeta^{-k})\cos\psi & 0 & -\beta (1-\zeta^{-k})\sin\psi \\ -\beta (1-\zeta^{k})\cos\psi & 4\alpha & \beta (1-\zeta^{k})\sin\psi & 0 \end{array}\right]\;. \end{array}$$

**Figure 2:**
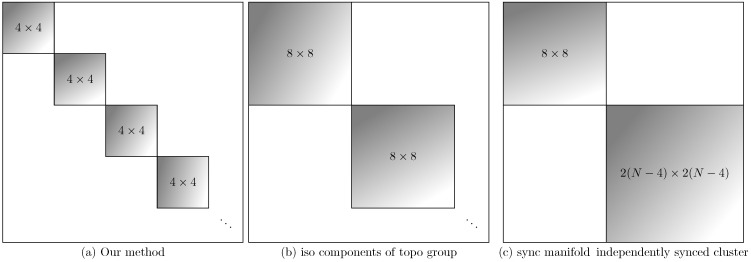
Guaranteed zero block structure of Jacobian matrix in various coordinates. Nonzero entries may occur only in the grayscale blocks. The coordinates are given by the following methods: (**a**) Isotypic components of $\sigma_{\mathrm{rot}}^{2}\sigma_{\pi }$ (via method introduced here). (**b**) Isotypic components of $\sigma_{\mathrm{rot}}^{4}$ (via method of Ref. [10]), and (**c**) Synchronization manifold and independently synchronized cluster set (via method of Ref. [2]).

Importantly, this coordinate system is independent of system state, within the solution set of interest (Equation (5)). The *V*’s used to compute each block consist of constants, and the resulting Jacobian block depends only on the phase difference ψ. Figure 2 shows the block structure, along with the coarser block structure predicted by alternate methods that consider purely-structural symmetries.

Instead of phase–amplitude oscillators, consider a ring of phase-only oscillators. Then, the decoupled state specified in Equation (5) is a guaranteed solution for $D_{4M}$ networks of identical nodes. Each has a T phase symmetry, so long as the coupling function respects a parity condition: g(ψ)+g(π−ψ)=0. However, the diagonal elements of the Jacobian are proportional to this term. This means that the Jacobian has zero trace. Since the trace is the sum of eigenvalues, any linearly stable mode implies at least one linearly unstable mode. And, so the decoupled state is not stable for phase-only oscillators.

Achieving a linearly-stable decoupled state for phase-only oscillators requires introducing long-range, nonpairwise, or nonphase coupling. Thus, although the amplitudes of the phase–amplitude oscillators have a fixed point at unity, the amplitude degree of freedom plays a central role in stability.

### 5.1. Uniform Frequencies: $\omega_{j}=\omega$

With uniform frequencies, the phase difference ψ between decoupled clusters is constant and the Jacobian is fixed in time. The decoupled state’s stability is thus given by the real parts of the Jacobian’s 2N eigenvalues. This problem then reduces to finding eigenvalues of its 2M blocks each of size 4×4. This requires finding the roots of quartic polynomials, which are:

(9) $$\begin{array}{c} \lambda =-\frac{1}{4}\pm \frac{1}{4}\sqrt{1-8\beta^{2}\left(1-\cos\frac{k\pi }{M}\right)\pm 4\beta \sqrt{2\left(16\alpha^{2}\cos^{2}\psi -\sin^{2}\psi \right)\left(1-\cos\frac{k\pi }{M}\right)}} \end{array}$$

The eigenvalues of Equation (7)’s linear dynamics are given by Equation (9), for k=0,1,…,2M−1 and with the two ± options taken independently.

Note that there are four possible, adjacent phase differences in Equation (5): ψ,π−ψ,π+ψ,2π−ψ. Eigenvalue λ’s dependence on ψ arises only through $\sin^{2}$ and $\cos^{2}$, which necessarily are equal for all four possible phase differences. This supports the physical equivalence of these values of ψ.

**Figure 3:**
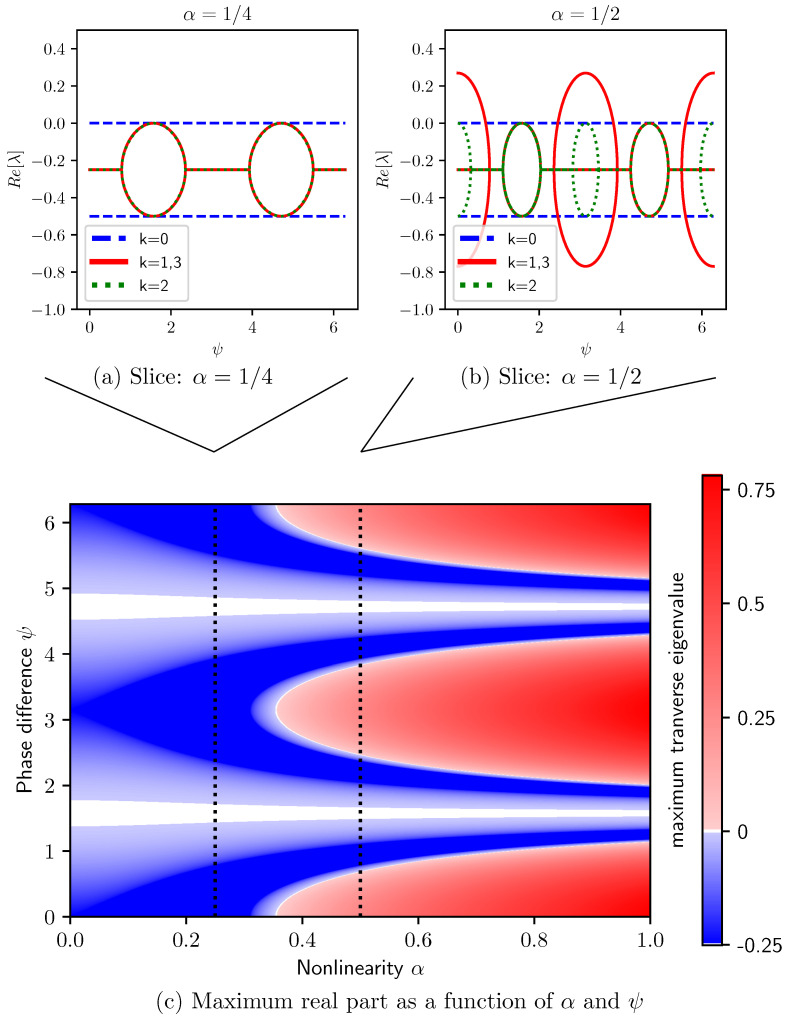
Numerical exploration of stability for uniform frequencies, $\omega_{j}=\omega$, at fixed coupling β=1. Panels (**a**,**b**) show the real part of $D_{k}$’s eigenvalues, Equation (9), versus phase difference ψ for two choices of Duffing coefficient α. Panel (**c**) shows the maximum real part of eigenvalues of blocks $D_{k\neq 0}$, quantifying the least stable perturbation that breaks the state’s symmetry as a function of ψ and α.

Figure 3a,b show the real part of $D_{k}$’s eigenvalues, obtained from Equation (9) for N=8 and β=1, as a function of phase difference ψ and for different values of nonlinearity: (a) α=1/4 and (b) α=1/2. Each *k*-block gives four eigenvalues, symmetric around −1/4 as expected from Equation (9). The blue dashed lines at Re(λ)=0 show neutral stability within the k=0 block. These eigenvalues are two-fold degenerate and define the torus of solutions (i.e., the class of trajectories) of interest. The transverse perturbations, k≠0, exhibit neutral stability at ψ={π/2,3π/2} at both values of nonlinearity. The instabilities in the larger nonlinearity are centered at ψ={0,π}, where there is a second pair of regions in which the outer square root of Equation (9) exhibits a real part.

Excluding the k=0 block, Figure 3c shows the maximum real part of $D_{k}$’s eigenvalues for N=8 and β=1 as a function of both phase difference ψ and nonlinearity α. This highlights the state’s largest instability at that point, with red being unstable, blue stable, and white linearly neutral. Here, we see the steady neutral stability at ψ={π/2,3π/2}, but we also see the instabilities growing from Re(λ)=−1/4 at ψ={0,π}, as α increases. From Figure 3b, we know that these unstable bands are in the k=1,3 block.

Due to the block diagonal structure induced by this state’s symmetries, we can precisely identify instabilities on the decoupled state: Equation (9) directly relates parameter values with the growth and decay of perturbations. All of the results presented in Figure 3 are calculated as closed-form expressions, capturing the eigenvalues of the 16×16 Jacobian matrix.

### 5.2. Alternating Frequencies, $\omega_{j}=\omega \mp \Omega /2$

In the experimental study of nanoelectromechanical oscillator networks [14] parameters α and β can be precisely controlled. While the $\omega_{j}$’s can be tuned quite close to one another, small deviations exist that break the $D_{4M}$ symmetry. When stable, all perturbations transverse to the fixed-point subspace are exponentially restored, and small dispersion in natural frequencies introduces a linear drift within the invariant subspace. These new drifting states remain within the space swept out by the uniform frequency states—sweeping θ and ψ in Equation (5)—but support a drifting phase difference ψ.

We capture this behavior theoretically by alternating the natural frequencies along the ring: $\omega_{j}=\omega \mp \Omega /2$. The form of Equation (5) remains a solution to Equation (2), but neighboring phase differences now have a well-defined drift that depends on the magnitude of the natural frequency difference between neighboring nodes: dψ/dt=Ω. Although the full system symmetry has changed, the solutions of interest respect the same symmetries—the group generated by $\sigma_{\pi }\sigma_{\mathrm{rot}}^{2}$—as in the uniform frequency case. This leads to the same block diagonalized Jacobian: Equation (8).

The Jacobian is now time-periodic. And, this requires Floquet theory for stability analysis, evolving each of a set of vectors for one whole period. By choosing this set as a basis for tangent space, we build the monodromy matrix—the linear map corresponding to evolution of perturbations through a period. Matrix eigenvalues capture how the perturbations evolve. The associated Floquet exponents are the natural logarithm of the magnitude of its eigenvalues, with positive values implying unstable growth and negative values showing stable decay. If the Jacobian were constant (i.e., periodic with any stated period), this procedure returns the real parts of the Jacobian eigenvalues themselves, as desired.

We performed the integration using the standard fourth-order Runge–Kutta scheme with 1000 integration steps [22]. The Jacobian’s diagonal form allows each 4×4 block to be integrated independently, significantly reducing the computational overhead for arbitrarily large oscillator rings.

**Figure 4:**
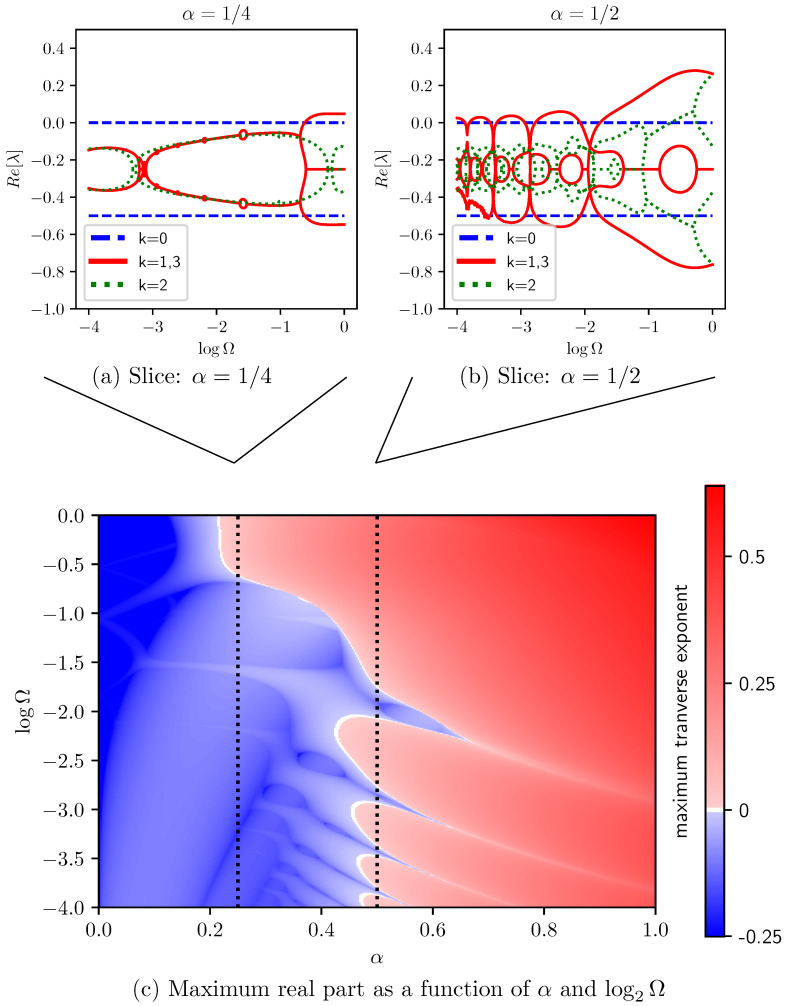
Numerical exploration of stability for alternating natural frequencies $\omega_{j}=\omega \mp \Omega /2$ with fixed coupling β=1: Panels (**a**,**b**) plot the real part of the Floquet exponents of $D_{k}$ (Equation (8)) versus relative frequency Ω for two choices of Duffing coefficient α. Panel (**c**) shows the maximum real part of $D_{k\neq 0}$’s Floquet exponents, tracking the least stable perturbation that breaks the state’s symmetry as a function of $\log_{2}\Omega$.

We find that the stability of the resulting state depends intricately on system parameters, including the natural frequency difference between adjacent oscillators, as shown in Figure 4. (Noisy results at low Ω in Figure 4b are a numerical artifact due to long integration times and nonzero Floquet exponents.) Rather than being a function of phase difference, stability is now a function of rate Ω at which the system drifts through phase differences.

Here, we see many overlapping bands of instability emerging from Re(λ)=−1/4. A series of such bands, going unstable around α=0.45, appear to again come from the k=1,3 blocks and leave small windows of stability—narrow ranges of Ω in which the state is stable.

Even though alternating frequencies in the oscillator ring break the system symmetry and induce a time-periodic Jacobian, the symmetries of the state itself are unchanged and the group-theoretic block-diagonalization is remains valid. This allows the stability analysis to be performed on four dimensional subspaces, even for arbitrarily large rings.

## 6. Discussion

We demonstrated how phase-shift symmetries in the nodes and the dynamics, together with symmetries in the network connectivity, can simplify stability calculations. This extends recent results on cluster synchronization to include symmetries beyond the node-connectivity automorphism group. We then used this approach to analyze the stability properties of attractors that emerge in a ring of phase–amplitude oscillators. We identified a dynamically decoupled state as particularly novel, showing that it is stable for a ring a phase–amplitude oscillators, but not linearly stable for the analogous ring of phase- only oscillators.

Our results here highlight the importance of oscillator amplitudes in generating polyadic and long-range effective coupling. A reduced phase equation for the NEMS dynamics is introduced in [14] but, to first order in coupling, it only includes the nearest-neighbor phase interactions. This illustrates the need for future analysis that either formally moves to higher-order phase models or retains anharmonicity via amplitudes.

Our methodology and results apply directly to a variety of similar models, including any anharmonic oscillator with an attracting oscillation-amplitude, such as Stuart–Landau oscillators [21]. The equivalence of the decoupled state to a fixed-point subspace is, in such cases, a result of linear coupling. That said, the coupling could have real rather than (or in addition to) imaginary coefficient iβ→K+iβ. This would directly change the steady-state amplitudes rather than their frequencies. When K>1, this leads to amplitude death. While the Jacobian block diagonalization is ambivalent to coupling type, the precise stability results do not immediately translate to systems where the coupling coefficient has a real component.

## Data Availability

The data presented in this study are available upon reasonable request to the corresponding author.
